# MK2 Inhibition as a Novel Treatment for Fibrosis in Primary Sclerosing Cholangitis via an IL-22-Dependent Mechanism

**DOI:** 10.3390/cells14131031

**Published:** 2025-07-05

**Authors:** Cody S. Howe, Ellen J. Beswick

**Affiliations:** Division of Digestive Diseases and Nutrition, Department of Internal Medicine, University of Kentucky, Lexington, KY 40506, USA

**Keywords:** primary sclerosing cholangitis, PSC, fibrosis, Mapkap2, MK2, IL-22

## Abstract

Primary sclerosing cholangitis (PSC) is a chronic liver disease characterized by bile duct inflammation and fibrosis, leading to cirrhosis and liver failure. Current therapies are limited to symptom management, with no approved treatments targeting fibrosis. We have identified the MAP kinase-activated protein kinase 2 (MK2) pathway as a potential therapeutic target for treating PSC due to its role in promoting inflammatory cytokine production and activation of fibroblasts. Thus, MDR2 knockout mice were treated therapeutically with MK2 inhibitors, which led to significantly reduced hepatic inflammation and fibrosis. Liver enzymes, collagen 1A1, and fibronectin were decreased in serum with MK2 inhibitor treatment. Furthermore, the production of IL-6, TNFα, CXCL5, collagen 1A1, and fibronectin was decreased in liver tissues and liver stellate cells, whereas the production of IL-10, G-CSF, and IL-22 was increased. MDR2KO mice treated with IL-22 also showed improvements in inflammation and fibrosis, along with increased IL-10 and G-CSF production. Taken together, we identified both a direct mechanism of MK2 regulation of fibrotic factors and an indirect cytokine-mediated mechanism whereby the levels of IL-22, IL-10, and G-CSF were increased with MK2 inhibition and contributed to decreased levels of fibrotic factors. These data suggest that the MK2 pathway is a promising treatment target for PSC.

## 1. Introduction

Primary sclerosing cholangitis (PSC) is a chronic liver disease that affects bile ducts of all sizes, including small interlobular ducts as well as larger intrahepatic and extrahepatic ducts. Chronic inflammation and fibrosis ultimately lead to liver damage and scarring of the bile ducts, leading to narrowing or blockage, which traps bile in the liver, leading to further liver damage [1]. Up to 75% of PSC patients also have inflammatory bowel disease, making it particularly challenging to treat. PSC is generally refractory to immunosuppressive therapy and lacks effective medical treatments that target the underlying pathophysiological mechanisms [2]. Consequently, disease progression is typical and often progresses to a stage that requires liver resection or transplantation as the only definitive treatment option. However, disease recurrence in the transplanted liver is not uncommon, with recurrent PSC estimated to occur in approximately 20% of patients [3]. Together, these challenges highlight the urgent need for therapeutic strategies that effectively target the inflammatory and fibrotic components of PSC pathogenesis.

Inflammation in PSC is driven by a complex interplay of immune, microbial, and biliary factors, resulting in injury to the bile ducts. Increased concentrations of pro-inflammatory cytokines, including IL-17A, IL-6, TNFα, and IFNγ, have been observed in patients with PSC [4,5], indicating an inflammatory environment driven by both innate and adaptive immune responses. As inflammatory cytokines/chemokines are produced, there is an influx of immune cells to the liver, where they become activated and exacerbate inflammation and fibrosis. A hallmark of PSC is also the aberrant recruitment of gut-homing lymphocytes to the liver, facilitated by hepatic expression of adhesion molecules and chemokines, thus linking intestinal immune dysregulation to hepatic inflammation [6]. Furthermore, it has been shown in one study that the numbers of neutrophils and T cells are highly increased in PSC and that their interaction is critical to disease progression [7]. Chronic inflammation is known to drive periductal fibrosis, with contributions from multiple hepatic cell types; however, the precise mechanisms linking chronic inflammation and fibrosis remain elusive.

In addition to immune cells, several other liver cell types are critical for fibrosis. Hepatic stellate cells are key contributors to liver fibrosis, where during liver damage, they differentiate into contractile, proliferative myofibroblasts with pro-inflammatory and fibrogenic properties [8]. Portal fibroblasts represent another important source of myofibroblasts, particularly in PSC. Here, liver injury also initiates the activation and differentiation of quiescent portal fibroblasts into myofibroblasts, a process that is further amplified by signals from cholangiocytes [9]. When cholangiocytes become reactive, they proliferate and secrete co-stimulatory molecules, chemokines, and pro-fibrogenic mediators that further stimulate fibrogenesis [10]. Additionally, bile acid accumulation due to cholestasis may exacerbate fibrosis by directly activating myofibroblasts or causing hepatocyte injury that perpetuates the inflammatory cascade [11]. Collectively, these immune and fibrotic mechanisms demonstrate the complexity of PSC pathogenesis and suggest that, given the limited efficacy of immunosuppressive therapies, targeting fibrogenic pathways may offer a more effective strategy for managing the pathological consequences of chronic inflammation.

We and others have identified the MK2 signaling pathway as a key regulator of inflammation [12,13]. MK2 is a stress-responsive serine/threonine kinase that plays a role in cytokine/chemokine production, such as IL-1β, IL-6, and TNF-α [14]. Although MK2 is a downstream target of p38 MAPK, MK2 exhibits lower activity under homeostatic conditions and can function independently of the much broader p38 pathway, making it a potentially safer and more selective target for therapeutic intervention [15]. The MK2 pathway may also promote fibrosis, as MK2 inhibition was shown by us to decrease fibrosis in inflammatory bowel disease (IBD) models [16] and by others in pulmonary and cardiac fibrosis [17,18]. Despite its well-established role in regulating cytokine production and emerging information on its impact on fibrosis, the specific contribution of MK2 to PSC pathogenesis remains unexplored.

In this study, we investigated the role of MK2 in the pathogenesis of PSC, focusing on its role in regulating inflammation and fibrosis. Using the MDR2-KO mouse model of PSC, we found that therapeutic administration of MK2 inhibitors after liver and bile duct damage occurred led to significantly reduced expression of pro-inflammatory cytokines and pro-fibrotic factors, along with decreased liver damage and fibrosis. Interestingly, MK2 inhibitor-treated mice also showed decreased expression of inflammatory cytokines, as expected, but led to the novel finding of elevated levels of IL-22, along with increased levels of IL-10 and G-CSF. To further explore this relationship, the mice were treated with exogenous IL-22 and displayed similarly reduced liver inflammation, fibrosis, and injury along with a similar increase in IL-10 and G-CSF. These findings suggest a potential antifibrotic role for IL-22 in PSC. Thus, MK2 inhibition may confer protection in PSC by dampening both inflammatory and fibrotic responses, in part through a cytokine-dependent mechanism.

## 2. Materials and Methods

### 2.1. Animals

MDR2KO (FVB.129P2-*Abcb4^tm1Bor^*/J) and wild-type mice were purchased from Jackson Laboratory (Bar Harbor, MA, USA) and bred in house. These mice were housed at the Animal Medical Facility, University of Kentucky, KY, USA, and were were housed in IVC rack cages under a 14/10 h light-dark cycle with access to standard chow pellets and drinking water ad libitum. The University of Kentucky Institutional Animal Care and Use Committee approved the mouse research protocols.

### 2.2. Animal Treatments

WT and MDR2KO mice were subjected to intraperitoneal injection of the MK2 inhibitor. When the mice were 12 weeks old, MK2 inhibitor (MK2i) (10 µg) treatment was given every other day (9 total injections) in 10 µL of DMSO or DMSO as a vehicle control. MDR2KO (FVB.129P2-*Abcb4^tm1Bor^*/J) mice were also subjected to intraperitoneal injection of recombinant mouse IL-22 (Fujifilm Irvine Scientific, Warminster, PA). When the mice were 12 weeks old, IL-22 (200 ng)treatment was given every other day (9 total injections) in 10 µL of sterile H_2_O or H_2_O as a vehicle control.

### 2.3. Cell Culture

The human LX-2 liver stellate cell line was obtained from MilliporeSigma (Burlington, MA, USA). LX-2 cells were cultured in Dulbecco’s modified Eagle’s medium (DMEM) (Gibco, Waltham, MA, USA) supplemented with 10% heat-inactivated fetal bovine serum (FBS) (Gibco, Waltham, MA, USA), 1% penicillin-streptomycin, and 1% L-glutamine (Invitrogen, Waltham, MA, USA) at 37 °C with 5% CO_2_ and 5% humidity. Cells were plated at 1.5 × 10^5^ and treated with 10ng/mL of TGF-b (Fujifilm, Warminster, PA, USA) for 24 h and then treated with 10µM of MK2 inhibitor or 10 ng/mL of either recombinant IL-22, IL-10, or G-CSF (Fujifilm, Warminster, PA, USA). Supernatants were collected for ELISA, and the cells were collected for gene expression analysis.

### 2.4. Serum Analysis

Mouse blood was drawn by cardiac puncture after euthanasia. C-reactive protein (CRP) was analyzed by Procarta Luminex bead assay (ThermoFisher Scientific, Waltham, MA, USA) according to the manufacturer’s instructions. Collagen 1 and fibronectin were measured by ELISA(Sigma-Aldrich, St. Louis, MO, USA). Aspartate aminotransferase (AST) and alanine transaminase (ALT) activity levels were measured in the serum of mice using a Piccolo Xpress blood chemistry analyzer (Abraxis BioScience, Los Angeles, California, USA).

### 2.5. Histopathological Analysis

Liver tissue sections were fixed in 10% paraformaldehyde and then transferred to ethanol. Fixed samples were sent to the Markey Cancer Center Biospecimen Procurement and Translational Pathology Shared Resource at the University of Kentucky. The tissues were then paraffin-embedded and stained according to a standard protocol for hematoxylin and eosin (H&E), Trichrome staining, and Sirius Red staining. Imaging of the stained liver sections was performed using the EVOS M5000 imaging system (ThermoFisher Scientific, Waltham, MA, USA).

### 2.6. RNA Extraction, cDNA Synthesis, and Real-Time PCR

Liver pieces were homogenized using a BeadBug™ 6 homogenizer (Benchmark Scientific, Sayreville, NJ, USA) in TRIzol™ reagent (ThermoFisher Scientific, Waltham, MA, USA). The human LX-2 liver stellate cell line was lysed directly with TRIzol™ reagent. RNA extraction was performed according to the manufacturer’s protocol. A NanoDrop™ Spectrophotometer was utilized to assess both the quality and quantity of the RNA. The total RNA (200 ng/µL) was subjected to reverse transcription using the High-Capacity cDNA Reverse Transcription Kit (ThermoFisher Scientific, Waltham, MA, USA) under the following PCR settings: 25 °C for 10 min, 37 °C for 120 min, and 85 °C for 5 min. Then, it was infinitely held at 4 °C. The quantification of mouse mRNA was performed using real-time PCR with validated FAM dye-labeled TaqMan^®^ probes (Applied Biosystems, Foster City, CA, USA) for *Actb*—Mm02619580_g1*, Acta2—*Mm00725412_s1, *Col1a1*—Mm00801666_g1, *Col1a2*—Mm00483888_m1, *Fap*—Mm01329177_m1, *Tnc*—Mm00495662_m1, and *Vim*—Mm01333430_m1. The reaction mixture consisted of cDNA, TaqMan^®^ Fast Advanced Master Mix (Applied Biosystems, Foster City, CA, USA), TaqMan^®^ Assays, and RNase-free water in a total volume of 10 µL. The quantification of human mRNA was performed using real-time PCR with validated FAM dye-labeled TaqMan^®^ probes (Applied Biosystems, Foster City, CA, USA) for *ACTB*—HS01060665_g1*, ACTA2—*HS00426835_g1*, COL1A2*—HS01028956_m1, *FAP*—HS00990791_m1, *FN1*—HS01549976_m1, *TNC*—HS01115665_m1, and *VIM*—HS00958111_m1. The reaction mixture consisted of cDNA, TaqMan^®^ Fast Advanced Master Mix (Applied Biosystems, Foster City, CA, USA), TaqMan^®^ Assays, and RNase-free water in a total volume of 10 µL. The real-time PCR cycle parameters consisted of an initial denaturation at 95 °C for 3 min, followed by 40 cycles of sequential incubations at 95 °C for 15 s and 60 °C for 1 min. The results were normalized to the expression of the housekeeping gene *Actb* (*ACTB* for humans). All experiments were performed on a QuantStudio™ 3 Real-Time PCR System (ThermoFisher Scientific, Waltham, MA, USA). The Ct parameter was defined as the endpoint used in real-time PCR quantification. The quantification of gene expression was performed using the comparative CT method, and the results are presented as the fold change relative to the mRNA level of the housekeeping gene.

### 2.7. Multiplex Array and ELISA Assays

Liver tissues harvested from MDR2KO and FVB mice were cut into 8 mg pieces (± 0.3 mg) and incubated in RPMI media (Corning, Tewskbury, MA, USA), supplemented with 10% fetal bovine serum (ThermoFisher Scientific, Waltham, MA, USA), 1% penicillin–streptomycin (Corning, Tewksbury, MA, USA), and 1% L – glutamine (ThermoFisher Scientific, Waltham, MA, USA) for 18 h. Supernatants collected from the liver samples were then used for Luminex multiplex arrays and ELISA (ThermoFisher Scientific, Waltham, MA, USA).

### 2.8. Statistical Analysis

All the statistical analyses were performed using GraphPad Prism 8.3.0 software. Unless otherwise indicated, differences between means were evaluated by one-way ANOVA for multiple comparisons and Student’s t test for the analysis of the significance between two groups. Values of *p* < 0.05 were considered statistically significant.

## 3. Results

### 3.1. Therapeutic Inhibition of MK2 in MDR2 KO Mice Inhibits Inflammation and Fibrosis

**Figure 1:**
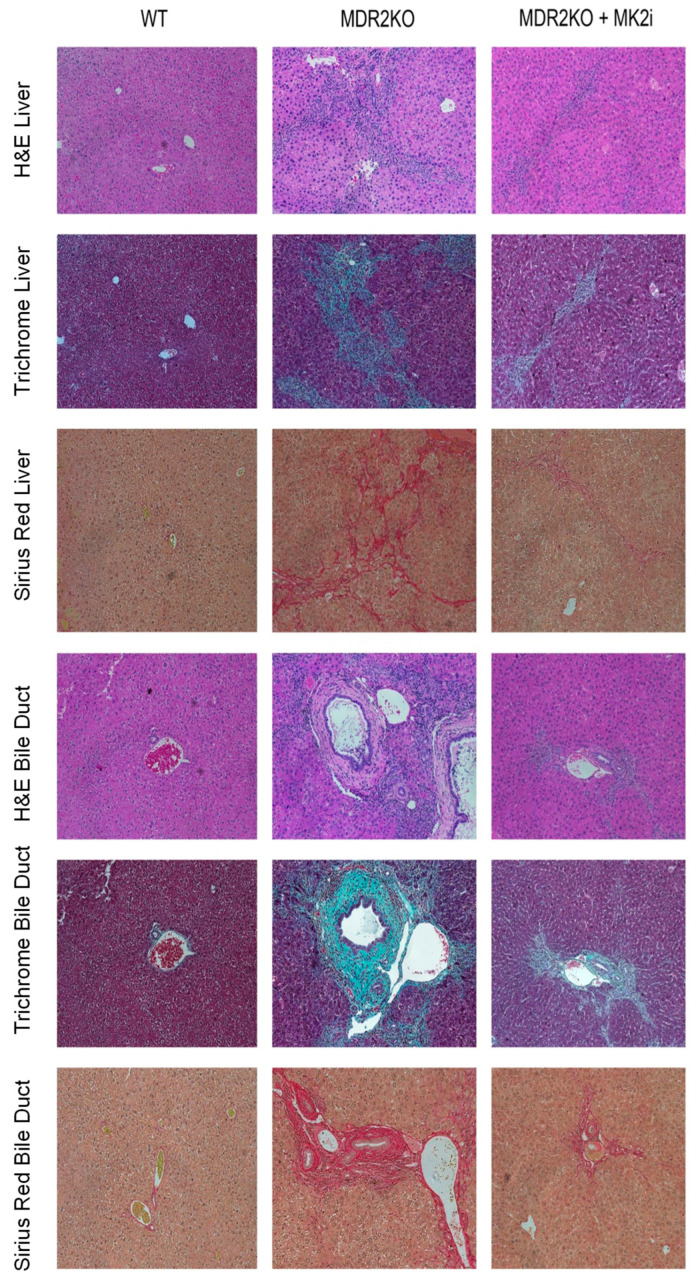
Therapeutic administration of MK2 inhibitors decreases inflammation and fibrosis in PSC. Histology of the liver and bile duct tissues from WT, MDR2 KO, and MDR2 KO mice treated with MK2i depict decreased inflammation, and fibrosis levels in the MDR2 KO mice treated with MK2i in both liver tissue and around bile ducts, as shown by H&E, Trichrome, and Sirius Red staining.

Since the MK2 pathway has been shown to regulate cytokine production, and we have recently suggested fibrosis-promoting activity in IBD [12,16], here, we sought to examine the impact of MK2 inhibition on PSC. MDR2 KO mice were utilized in order to evaluate the role of the MK2 pathway in inflammation and fibrosis of the liver and bile ducts and to test the potential of MK2 inhibitors to inhibit these processes. WT and MDR2KO mice at approximately 12 weeks of age, when fibrosis begins [19], were treated with 10 µg of the MK2 inhibitor three times a week for 3 weeks. Liver and bile duct tissue were examined by H&E staining, and an influx of immune cells was seen in the MDR2KO liver and bile duct tissues compared to WT mice (Figure 1). However, in mice treated with MK2 inhibitors, immune cells are decreased compared to MDR2 KO mice. Furthermore, tissues also underwent trichrome staining for collagen deposition as an indicator of fibrosis. As Figure 1 indicates, WT mouse liver and bile ducts show very little staining for collagen, while significant amounts are seen in MDR2-KO mice, but staining is decreased in MDR2 KO mice treated with MK2 inhibitors. To further confirm these results, Picro Sirius Red staining was also performed. As seen with Trichrome staining, Sirius Red was also increased in MDR2 KO tissues compared with WT tissues but decreased with MK2 inhibitor treatment (Figure 1). Example histology images are shown here to depict changes, while quantitative changes are shown below. These results suggest that MK2 inhibitors protect against inflammation and collagen deposition in liver and bile duct tissues.

### 3.2. Therapeutic Inhibition of MK2 in MDR2 KO Mice Inhibits the Production of Circulating Inflammatory and Fibrotic Factors

**Figure 2:**
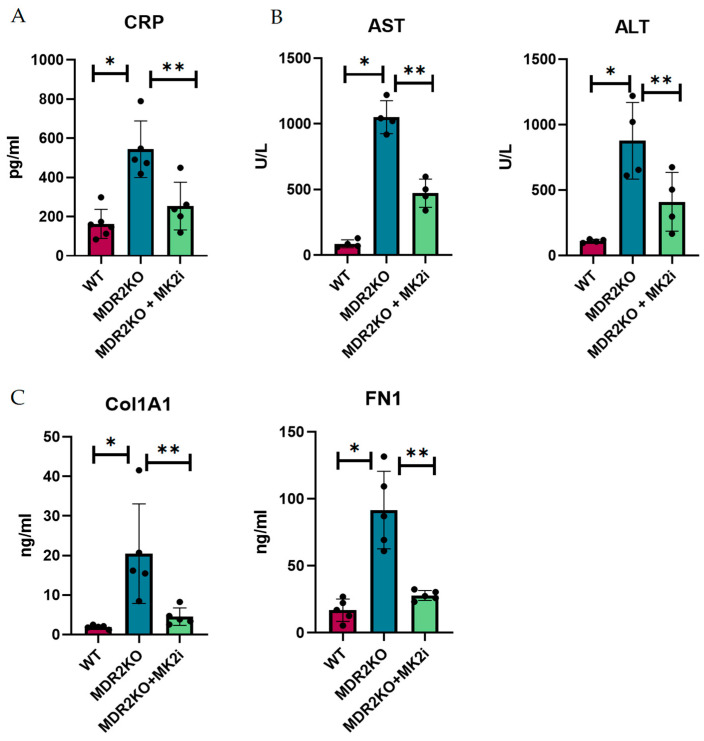
MDR2KO mice receiving MK2i have decreased levels of circulating inflammatory and fibrosis factors. Serum samples from WT-, MDR2 KO-, and MDR2 KO MK2i-treated mice were analyzed for inflammation and fibrotic factors, and compared with MDR2 KO mice, MDR2KO mice treated with MK2i presented significantly decreased levels of (**A**) CRP, (**B**) AST and ALT, and (**C**) Col1 and FN1. N = 5, *p* < 0.05 for * WT vs. MDR2KO and ** MDR2KO vs. MDR2KO + MK2.

Since histological analysis suggested that MK2 inhibition decreased the influx of immune cells and collagen deposition, we sought to determine the soluble factors involved in this process. Thus, the mouse serum from these experiments was examined for inflammatory and fibrotic factors. As a general measure of inflammation, CRP was measured by singleplex bead assay. CRP was increased by approximately 3-fold in MDR2 KO mice compared with WT mice but significantly decreased in MDR2 KO mice treated with MK2 inhibitors (Figure 2A). As a measure of liver function that is used clinically, AST and ALT were examined in serum (Figure 2B), where both were increased in MDR2KO mice but decreased with MK2 inhibitor treatment. The serum levels of fibronectin and collagen 1A1, which are indicators of fibrosis, were also examined by ELISA and were shown to be increased in MDR2-KO mice compared with WT mice and significantly decreased in mice treated with MK2 inhibitors (Figure 2C).

### 3.3. Therapeutic Inhibition of MK2 in MDR2 KO Mice Inhibits Production of Tissue-Associated Inflammatory and Fibrotic Factors

**Figure 3:**
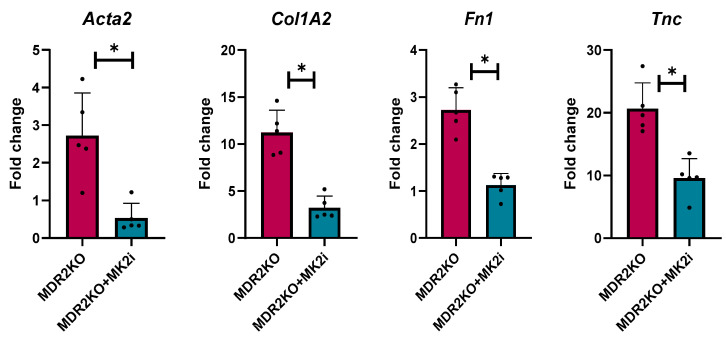
MDR2KO mice receiving MK2i have decreased fibrosis gene expression in liver tissues. Gene expression analysis by qRT PCR indicates that compared with WT mice, MDR2KO mice have increased *Acta2, Col1A2, FN1, and TNC* levels, which were substantially decreased with MK2 inhibitor treatment. N = 5, * *p* < 0.05 for MDR2KO vs. MDR2KO + MK2i.

As our initial serum analysis indicated a decrease in the levels of circulating fibrotic factors, we further examined the gene expression of several genes critical for fibrosis related proteins in mouse liver tissues. Gene expression was analyzed for alpha smooth muscle actin (*αSma*), collagen 1A2 (*Col1A2*), fibronectin (*FN1*), and tenascin-C (*Tnc*) expression. Compared with WT control mice, MDR2KO mice had significant increases in the expression of these four genes. However, the expression of these genes was significantly lower in the mice treated with the MK2 inhibitor than in the WT control mice (Figure 3). These data further suggest a protective effect of MK2 inhibition on liver fibrosis by decreasing fibrotic gene expression.

**Figure 4:**
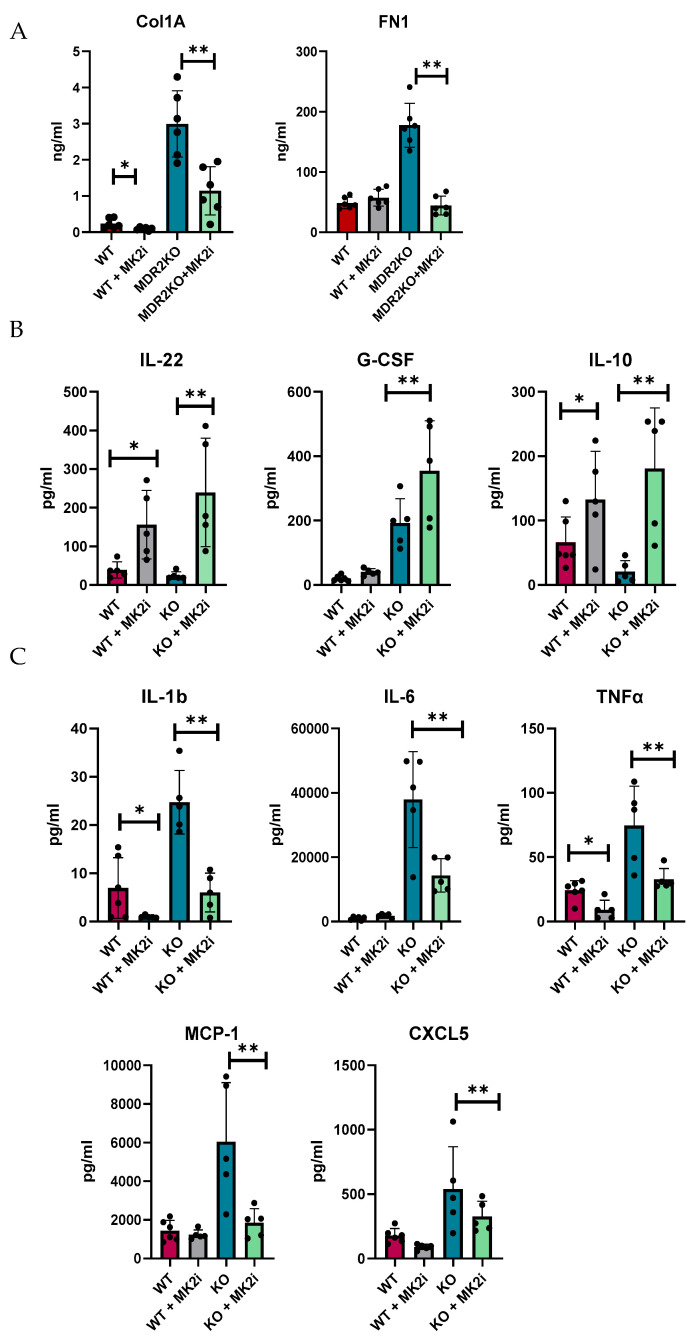
MDR2KO mice receiving MK2i have decreased levels of fibrotic factors and pro-inflammatory cytokines and a concurrent increase in the levels of anti-inflammatory cytokines. Supernatants from 8 mg of liver tissue were analyzed by (**A**) ELISA for collagen 1 and fibronectin and by multiplex arrays for (**B**) cytokines whose levels were increased with MK2i and (**C**) pro-inflammatory cytokines whose levels were decreased with MK2i. N = 5–6, * *p* < 0.05 for WT vs. MDR2KO and ** *p* < 0.05 for MDR2KO vs. MDR2KO + MK2i.

We and others have shown that MK2 is a major regulator of inflammatory cytokines [20,21,22,23]; thus, we examined the production of cytokines in murine liver tissues by multiplex bead array. Tissues were divided into 8 mg pieces and incubated for 18 h in complete RPMI media. Supernatants were collected and analyzed for soluble fibrotic factors. Fibronectin and collagen 1A1 were also found to be increased in supernatants of liver tissues of MDR2-KO mice compared with those of WT mice, but factors were decreased in the tissues of the mice that received MK2 inhibitor treatment (Figure 4A). Furthermore, the protective cytokines IL-22, G-CSF, and IL-10 were found to be increased in supernatants from liver tissues from MK2 inhibitor-treated mice (Figure 4B). These cytokines may have anti-inflammatory and tissue repair properties, but their potential impact on fibrosis needs to be determined. Concurrently, inflammatory cytokines were increased in MDR2KO mouse liver tissues compared with WT mouse liver tissues and decreased in supernatants from tissues incubated with MK2 inhibitors (Figure 4C). WT mouse livers incubated with MK2i did not significantly differ from those of the control group, suggesting that the MK2 pathway is specific to an inflammatory/fibrotic state. These data suggest that both circulating and liver tissues are decreased in profibrotic factors upon MK2 inhibition.

### 3.4. Liver Stellate Cell Regulation of Fibrotic Factors Is Dependent on MK2 and Downstream Cytokines

**Figure 5:**
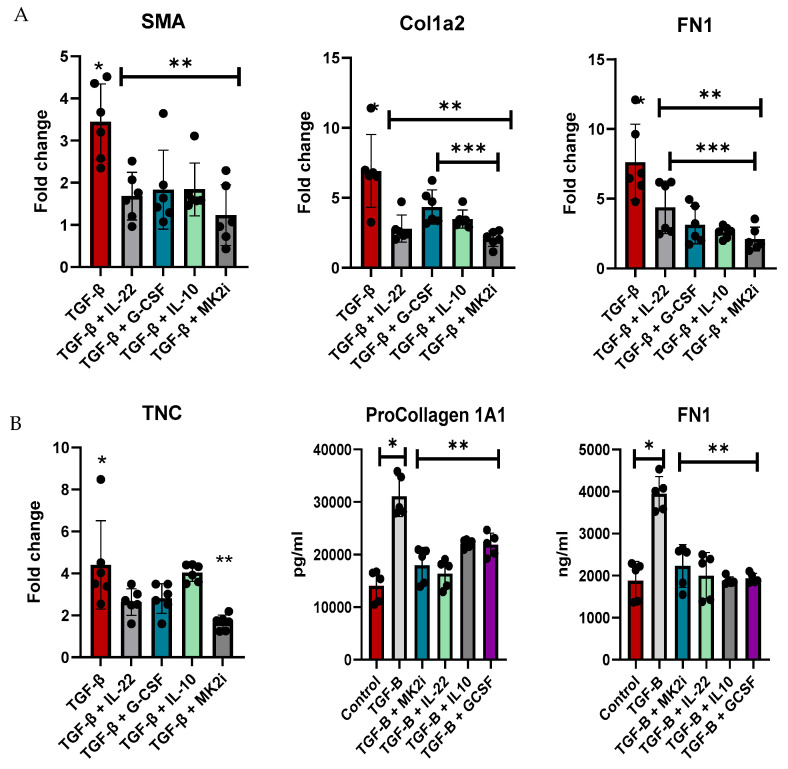
LX-2 human liver stellate cells express increased levels of fibrosis factors upon TGF-β treatment, which are decreased with cytokine and MK2i treatment. LX-2 cells treated with TGF-β show increased (**A**) gene expression of fibrotic genes and (**B**) production of procollagen 1A1 and fibronectin in supernatants, which decreased with IL-22, G-CSF, IL-10, or MK2i treatment. N = 6, * *p* < 0.05 for LX-2 vs. LX-2 + TGFβ, ** *p* < 0.05 for LX2 + TGF-β vs. LX2 + TGF-β + cytokines or MK2i, and *** *p* < 0.05 for LX-2 + TGFβ + cytokines vs. LX-2 + TGFβ + MK2i.

Normal human liver stellate cells were utilized to examine the direct regulation of fibrotic factor gene expression and the secretion of soluble factors. LX-2 cells were treated with TGF-β for 48 h to increase the expression of fibrotic factors and then treated with MK2 inhibitors or cytokines that were increased during MK2 inhibitor treatment, which included IL-22, G-CSF, and IL-10, for 24 h. Cells were examined for fibrotic gene expression, where we found *α-Sma, Col1A1, and Fn1* to be significantly decreased by cytokine treatment and MK2i treatment (Figure 5A). MK2i treatment also significantly decreased *Col1A1* compared with G-CSF and IL-10 treatment *and Fn1* compared with IL-22 treatment. *TNC* expression was decreased by MK2i treatment but not by cytokine treatment.

Supernatants were analyzed for fibronectin and pro-collagen 1A1, which are markers of fibrosis that are increased in mouse serum. Compared with control cells, cells treated with TGF-β showed significantly increased soluble fibrotic factor levels, but these levels were decreased by MK2 inhibitor treatment (Figure 5B). Furthermore, treatment of cells with IL-22, G-CSF, or IL-10 inhibited the production of these soluble factors. Taken together, our data indicate that MK2 inhibition decreases fibrotic gene expression and soluble factors, which are regulated by cytokines whose expression is upregulated during MK2 inhibition, thus suggesting a cytokine-mediated mechanism regulating liver fibrosis.

### 3.5. IL-22 Decreases Inflammation and Liver Fibrosis in Mice

**Figure 6:**
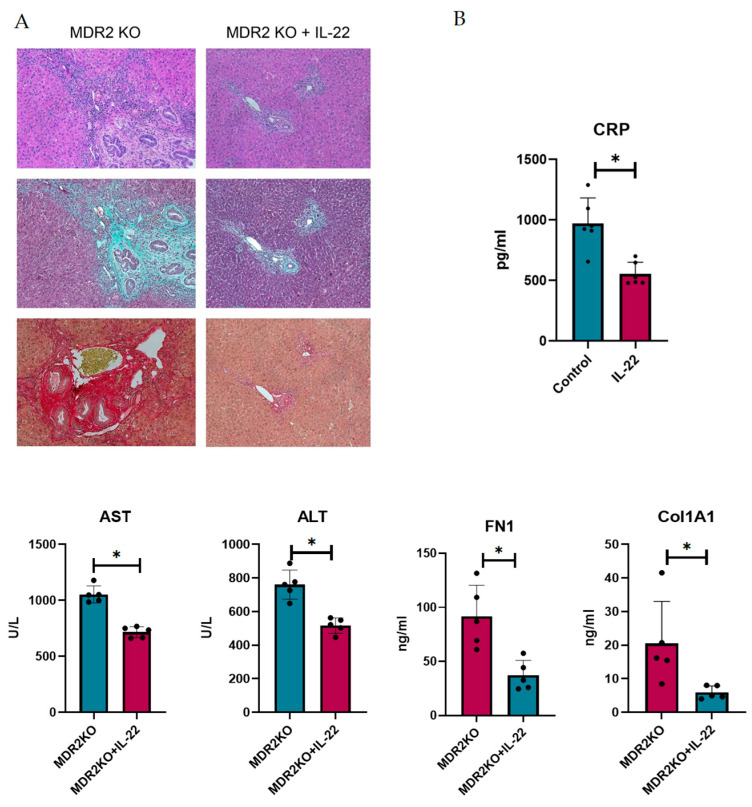
IL-22 treatment decreases inflammation and fibrosis in mouse PSC. (**A**) Histology of liver and bile duct tissues from MDR2 KO mice and MDR2 KO mice treated with IL-22 revealed decreased inflammation and fibrosis levels in mice treated with IL-22. (**B**) Serum from these mice also showed decreased CRP, AST, ALT, Col1A1, and FN1 levels. N = 5, * *p* < 0.05 for MDR2KO vs. MDR2KO + IL-22.

IL-22 has been shown to play dual roles in inflammation and fibrosis and is suggested to be critical in liver repair. Thus, we sought to examine the potential for protection in PSC. To achieve this, MDR2 KO mice at approximately 12 weeks of age, when fibrosis has begun to develop, were treated with 200 ng of IL-22 three times a week for 3 weeks. Liver and bile duct tissues were examined for inflammation and fibrosis. As shown in Figure 6A, H&E staining suggests that IL-22 treatment decreases the influx of immune cells into liver tissues. Collagen deposition, as measured by Trichrome and Sirius Red staining, also appeared to be substantially decreased in the IL-22-treated mice (Figure 6A). Serum from the mice was analyzed for CRP to assess inflammation, which was significantly decreased, and AST and ALT liver enzymes were also decreased in the IL-22-treated mice (Figure 6B). Furthermore, the levels of circulating collagen 1A1 and fibronectin were also decreased (Figure 6B).

**Figure 7:**
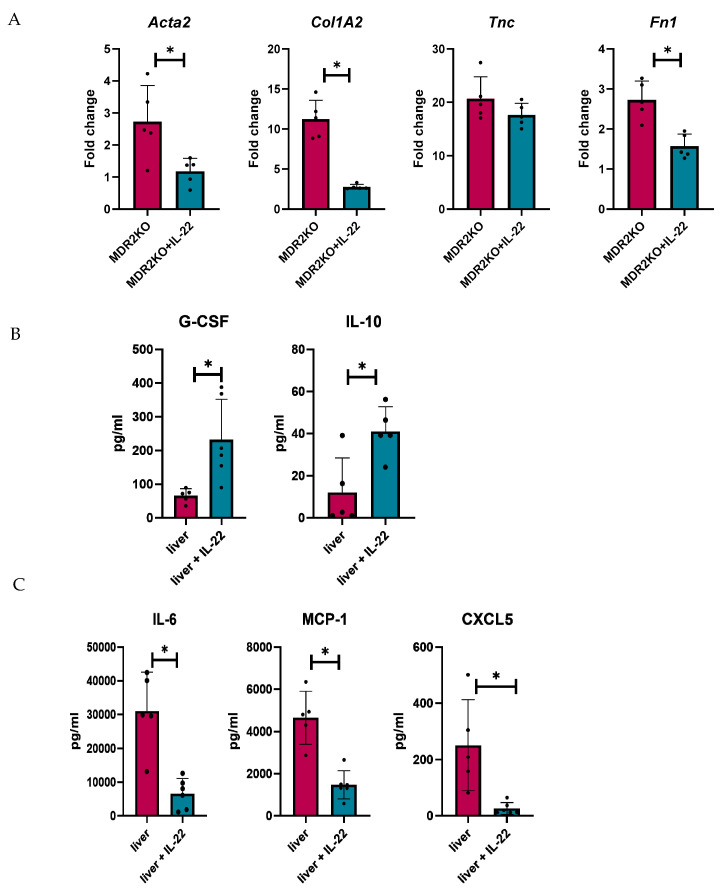
IL-22 treatment decreases fibrosis-related genes and the production of inflammatory cytokines in mouse PSC liver tissues. Analysis of livers from MDR2KO mice compared with those from MDR2KO mice treated with IL-22 show (**A**) decreased *Acta2, Col1A2, and FN1* but not *TNC* gene expression and (**B**) increased G-CSF and IL-10 but (**C**) decreased IL-6, MCP-1, and CXCL5 levels in the supernatants by multiplex array. N = 5, * *p* < 0.05 for MDR2KO vs. MDR2KO + IL-22.

Fibrosis genes were also analyzed in tissues from these mice, where *α-Sma, Col1A1, and Fn1* were significantly decreased in IL-22-treated MDR2KO mice compared with control mice (Figure 7A), but *Tnc* was not decreased, similar to the results in fibroblast cells in Figure 5. Tissue cytokine and fibrotic factor production were also examined in the tissue supernatants by multiplex bead array and ELISA, respectively. IL-22 treatment was found to decrease the levels of IL-6, MCP-1, and CXCL5 in MDR2 KO mouse livers (Figure 7B), which was also found in MK2 inhibitor-treated mice, as shown in Figure 3. Interestingly, we found increases in G-CSF and IL-10 in IL-22-treated mouse livers (Figure 7C), suggesting a collaborative role for these cytokines in regulating inflammation and fibrotic factors.

## 4. Discussion

The MK2 pathway is known to regulate inflammatory cytokine production. In this study, we investigated MK2 as a critical signaling regulator that modulates key aspects of PSC pathogenesis. Using the MDR2 KO mouse model, which mimics the histological and immunological features of human PSC, we showed that the inhibition of MK2 significantly reduced liver inflammation and fibrosis. These results are consistent with the established roles of MK2 in regulating cytokine and chemokine production, including IL-1β, IL-6, and TNF-α, in other models of chronic inflammation [24,25]. In addition to these known targets, we also observed reduced production of MCP-1 and CXCL5 with MK2 inhibition, which are important chemokines in immune cell recruitment during inflammation. Our group and others have previously reported that MK2 is capable of regulating MCP-1 [22,26]. However, to our knowledge, this is the first study identifying MK2-mediated modulation of CXCL5 expression. CXCL5 is a chemokine involved in neutrophil recruitment and angiogenesis [27,28]. Other studies have shown that CXCL5 is upregulated in various liver diseases and may play a role in hepatocellular carcinoma [29,30]. Our findings extend this knowledge by demonstrating that MK2 inhibition can modify these responses in the context of PSC, suggesting that MK2 may serve as a viable therapeutic target in this disease.

In addition to the expected suppression of pro-inflammatory factors, we observed increases in the expression of IL-22, IL-10, and G-CSF in MK2 inhibitor-treated mice. It is not clear if MK2 regulates these cytokines at the transcriptional level or indirectly through changes in overall cytokine signaling. These cytokines have been implicated in immunoregulatory and tissue-protective functions across multiple organ systems, including the liver [31,32,33]. IL-22 is a member of the IL-10 cytokine family and has been shown to support epithelial barrier integrity, induce antimicrobial peptide production, and stimulate tissue repair in models of liver injury [34,35]. Previous studies have indicated that IL-22 can promote liver regeneration and protect hepatocytes and cholangiocytes from injury through STAT3-dependent pathways, which leads to increased cell proliferation, reduced apoptosis, and improved antioxidant and anti-inflammatory responses [36]. In our study, exogenous IL-22 treatment recapitulated the protective effects observed with MK2 inhibition, including reduced fibrosis and liver damage, providing further evidence for its functional role in mitigating PSC-like pathology. The ability of IL-22 to modulate both inflammation and fibrosis warrants further investigation as a potential therapeutic target in PSC.

We also found the levels of G-CSF and IL-10 to be increased with both MK2i and IL-22 treatment. While the upregulation of IL-10 from inhibition of MK2 has been previously demonstrated by our group, another study suggested that MK2 is needed for the production of IL-10 [16,37]. IL-10 expression may depend on the inflammatory disease state, and our work focused on highly inflamed models. Previous studies in other models of liver inflammation have shown that IL-10 can reduce hepatic fibrosis, in part by limiting macrophage activation and altering T-cell function [32]. In a previous study, IL-10 KO mice exposed to chronic carbon tetrachloride developed significantly more liver fibrosis, further supporting that IL-10 may play a role in preventing hepatic fibrosis. G-CSF has been shown to promote liver regeneration by mobilizing hematopoietic stem cells, enhancing hepatic progenitor cell activity by increasing the proliferation rate, aiding in the progenitor’s ability to participate in liver regeneration, and improving survival in patients with decompensated cirrhosis [38,39,40]. However, a randomized, multicenter, controlled phase II trial study showed that G-CSF did not improve survival in patients with acute-to-chronic liver failure [41]. While the role of G-CSF in PSC has not been fully elucidated, its elevation in MK2 inhibitor-treated mice and IL-22-treated mice may reflect a general regenerative response to restore liver homeostasis.

To our knowledge, this is the first report linking MK2 inhibition to the upregulation of IL-22 and G-CSF in PSC liver tissues. IL-22 is a cytokine typically involved in maintaining mucosal barrier integrity and promoting epithelial regeneration [42,43]. In epithelial and stromal cells, IL-22 signals primarily through the STAT3 pathway; however, it can also activate MAPK cascades, suggesting a possible link with MK2, although direct interactions between the two have not been investigated [44,45]. The simultaneous upregulation of these cytokines following MK2 inhibition suggests a potential protective or therapeutic response that promotes decreased inflammation and the restoration of tissue integrity. Together, these findings suggest that MK2 inhibition may be used therapeutically to treat PSC in an indirect manner through the regulation of cytokines.

MK2 may also regulate fibrosis factors directly. This was shown in LX-2 liver fibroblasts, which were treated with TGFβ to upregulate the expression of the fibrotic factors collagen 1A1 and fibronectin, but this was reversed by MK2 inhibitor treatment at both the protein and gene levels. There have been other studies that have shown that MK2 plays a regulatory role in fibrotic responses by modulating fibroblast activity and extracellular matrix production in pulmonary fibrosis and mesenchymal cells in chronic inflammatory bowel disease models [46,47]. These findings further support that MK2 may play a role in the regulation of fibrotic factors.

This study is the first to examine the role of MK2 in liver/bile duct inflammation and fibrosis. MK2 may be a particularly valuable target since it regulates far fewer genes than some other signaling pathways that are targeted with inhibitors. MK2 inhibitor drugs have undergone early clinical trials for pulmonary and autoimmune diseases with few reported side effects [48,49,50], suggesting that they are promising new treatment approaches that should be further investigated [1,2,3]. Our study did not monitor long-term survival or liver function, so further studies are needed to investigate the treatment timeline and long-term effects of MK2 inhibition. In conclusion, we demonstrated that MK2 inhibition not only reduces hepatic inflammation and fibrosis but also promotes the expression of cytokines associated with tissue protection and immune regulation. These results support the continued investigation of MK2 inhibition as a promising treatment strategy for PSC and other fibrotic liver conditions.

## Data Availability

The datasets generated during and/or analyzed during the current study are available from the corresponding author on reasonable request.

## Abbreviations

The following abbreviations are used in this manuscript:

MK2	MAP kinase-activated protein kinase 2
PSC	Primary sclerosing cholangitis
MDR2	Multidrug resistance 2
IBD	Inflammatory bowel disease
MK2i	MK2 inhibitor
ALT	Alanine transferase
AST	Aspartate aminotransferase
H&E	Hematoxylin and eosin
